# Female sex bias in Iberian megalithic societies through bioarchaeology, aDNA and proteomics

**DOI:** 10.1038/s41598-024-72148-x

**Published:** 2024-09-23

**Authors:** Marta Díaz-Zorita Bonilla, Gonzalo Aranda Jiménez, Margarita Sánchez Romero, Rosa Fregel, Katharina Rebay-Salisbury, Fabian Kanz, Miriam Vílchez Suárez, Sonia Robles Carrasco, Paula Becerra Fuello, Alejandra C. Ordónez, Michael Wolf, Javier González Serrano, Lara Milesi García

**Affiliations:** 1 Institute for Pre- and Protohistory and Medieval Archaeology, University of Tübingen, Tübingen, Germany; 2 Department of Prehistory and Archaeology, University of Granada, Granada, Spain; 3 Department of Biochemistry, Microbiology, Cell Biology and Genetics, Universidad de La Laguna, San Cristóbal de La Laguna, Spain; 4 Department of Prehistoric and Historical Archaeology, University of Vienna, Vienna, Austria; 5 Austrian Academy of Sciences, Vienna, Austria; 6 Center for Forensic Medicine, Medical University of Vienna, Vienna, Austria; 7 Department of Historical Sciences, University of Las Palmas de Gran Canaria, Las Palmas de Gran Canaria, Spain; 8 Department of Analytical Chemistry, University of Vienna, Vienna, Austria; 9 Department of Historical Sciences, University of Malaga, Malaga, Spain

**Keywords:** Megalithism, Iberian Peninsula, Funerary ritual, Gender archaeology, Amelogenin peptide, Molecular sex, Human osteology, Proteomics, Archaeology, DNA

## Abstract

Uncertainties regarding traditional osteological methods in biological sex estimation can often be overcome with genomic and proteomic analyses. The combination of the three methodologies has been used for a better understanding of the gender-related funerary rituals at the Iberian megalithic cemetery of Panoría. As a result, 44 individuals have been sexed including, for the first time, non-adults. Contrary to the male bias found in many Iberian and European megalithic monuments, the Panoría population shows a clear sex ratio imbalance in favour of females, with twice as many females as males. Furthermore, this imbalance is found regardless of the criterion considered: sex ratio by tomb, chronological period, method of sex estimation, or age group. Biological relatedness was considered as possible sociocultural explanations for this female-related bias. However, the current results obtained for Panoría are indicative of a female-centred social structure potentially influencing rites and cultural traditions.

## Introduction

Biological sex estimation is one of the key aspects in the study of past populations. Traditional anthropological methods, such as those based on skeletal morphology and metrics, are often challenging. This is due to various aspects such as the state of preservation, dimorphism within a given population and the training and experience of bioarchaeologists^1^. Sexual dimorphism cannot be reliably estimated in non-adult individuals^2–4^ and for adults, the pelvis and skull present the most dimorphic features^5^. Ideally, when analysing single and well preserved skeletons, the combination of morphological and metrical methods would give us the most accurate sex estimation. However, this is not the case when human remains are found scattered, fragmented, mixed and poorly preserved. This is usually the case of megalithic tombs, which are comprised of multi-depositional ritual and mortuary events producing complex palimpsests^6–11^. Under these conditions, sex estimations can only be established in very few cases, which prevents any fine-grained discussion of palaeodemographic profiles and gender-related funerary rituals.

Recent methodological advances in proteomic and genomic techniques have led to a profound change in our perception of prehistoric societies. Sexual dimorphism can now be approached using two complementary and highly reliable sex determination methods based on the analysis of amelogenin peptides in tooth enamel^12–18^ and the study of ancient genomes^19,20^. In Iberia, ancient DNA (aDNA) analysis has expanded rapidly and focuses mainly on genetic ancestry that links the 3rd millennium cal BC Iberian societies with the expansion of steppe-pastoralists from Eastern Europe, as well as on the study of biological relatedness and kinship^21–25^. More recently, amelogenin peptide analysis has also begun to be applied to estimate biological sex at Iberian prehistoric sites^26–28^.

The Panoría megalithic cemetery offers an excellent opportunity for exploring gender-related aspects of funerary rituals. Meticulous recording techniques involving trained bioarchaeologists have produced a large bone assemblage that has been analysed from an osteoarchaeological point of view^11,29, 30^. The aim of this study is to provide a better understanding of gender-related funerary practices through fine-grained sex estimation. For this purpose, we explored a multi-proxy approach that combines genomic, proteomic and osteological data with detailed contextual archaeological features and radiocarbon chronology. For the first time, a comprehensive sex profile of an Iberian megalithic site includes both adult and non-adult individuals.

## Archaeological background: the Panoría cemetery

**Fig. 1:**
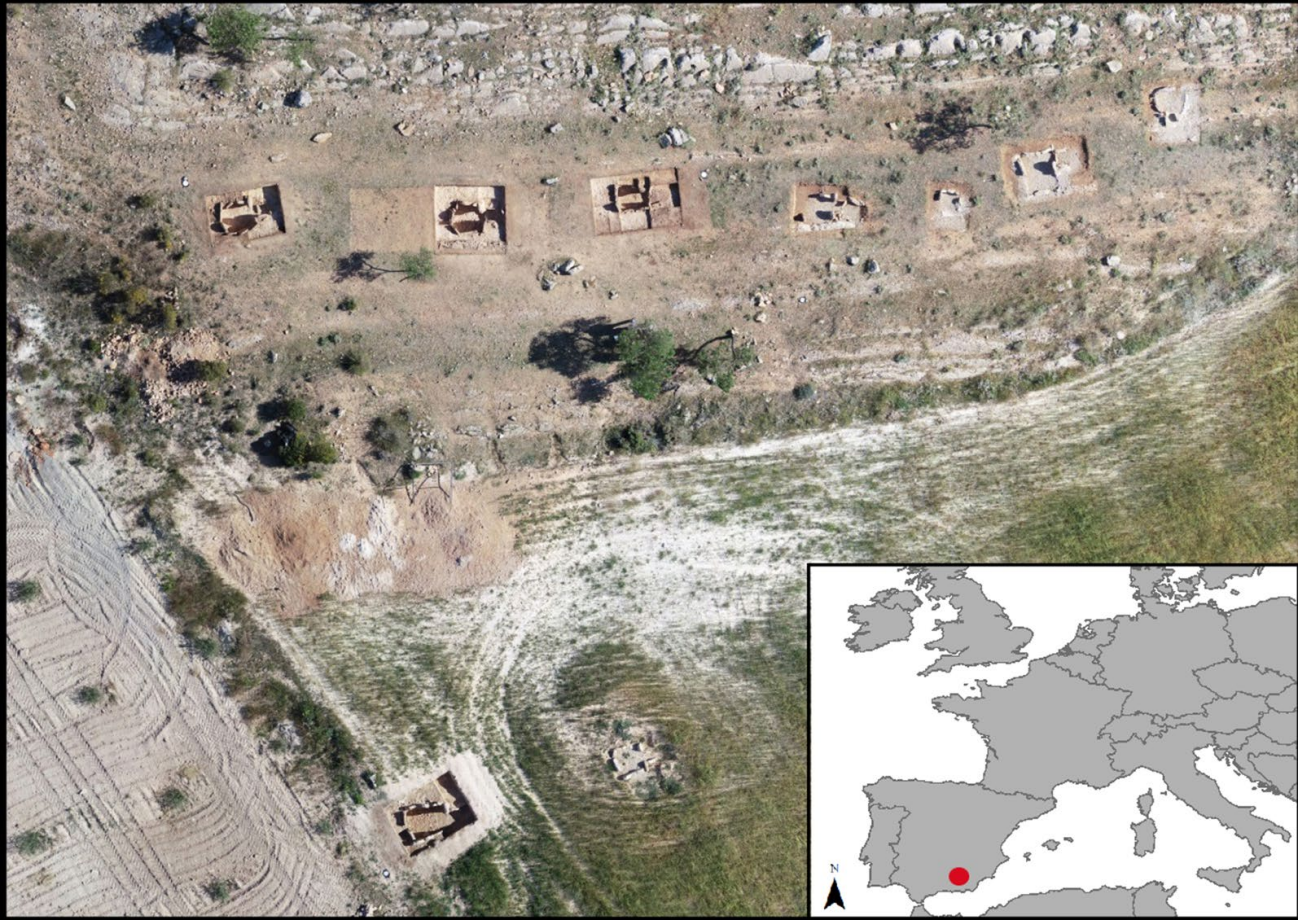
Orthophotography with the location of the 9 excavated tombs at Panoría cemetery. Up row, from left to right: graves 15, 3, 11, 10, 8, 7 and 6. Bottom row, from left to right: graves 17 and 18. This orthophotography was created by G.A.J. using a drone equipment (DJI Inspire-1) and processed with a SfM-based technology Agisoft-photoscan pro® 2.0.

**Fig. 2:**
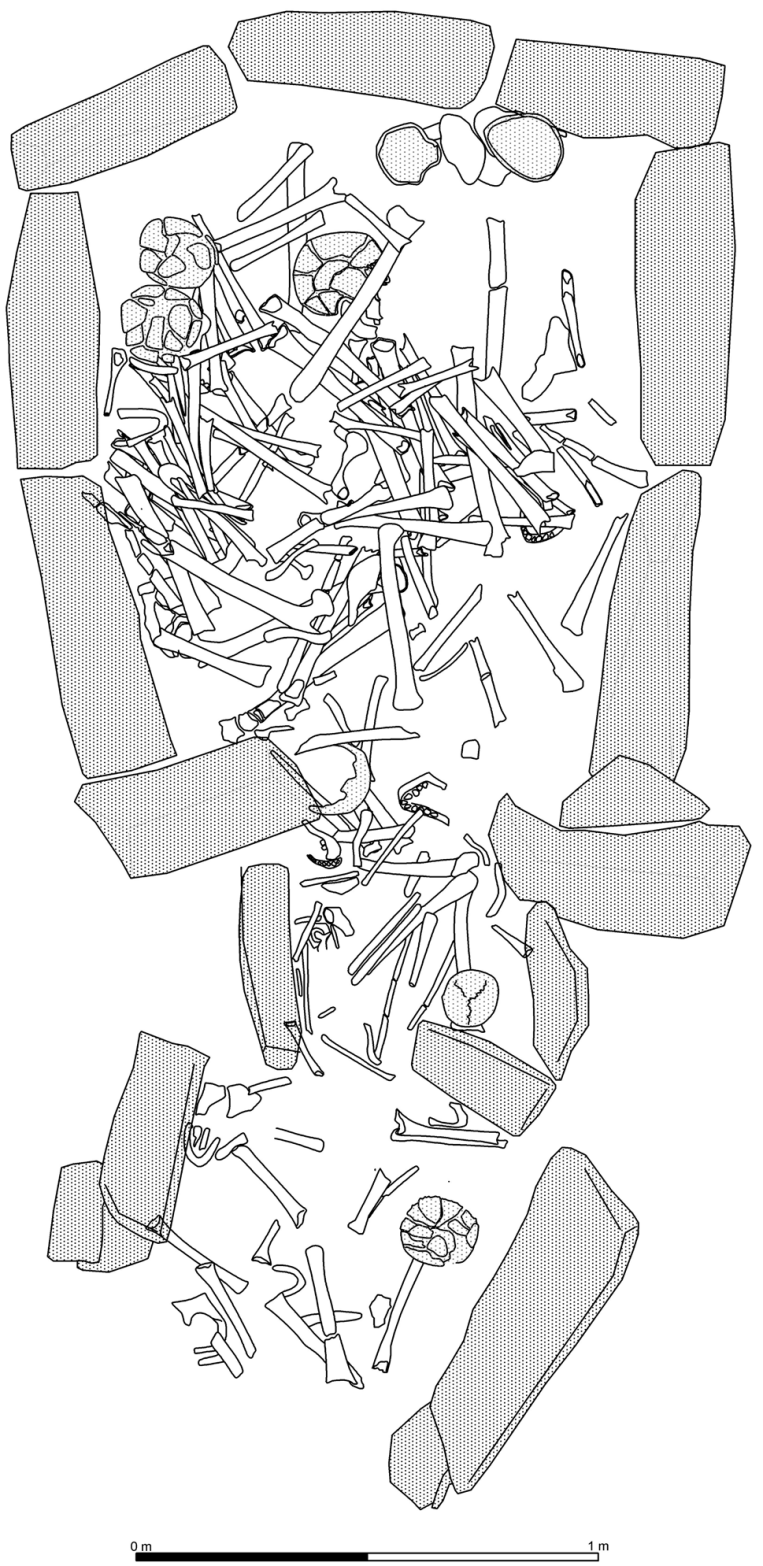
Human bone remains from Tomb 10.

**Fig. 3:**
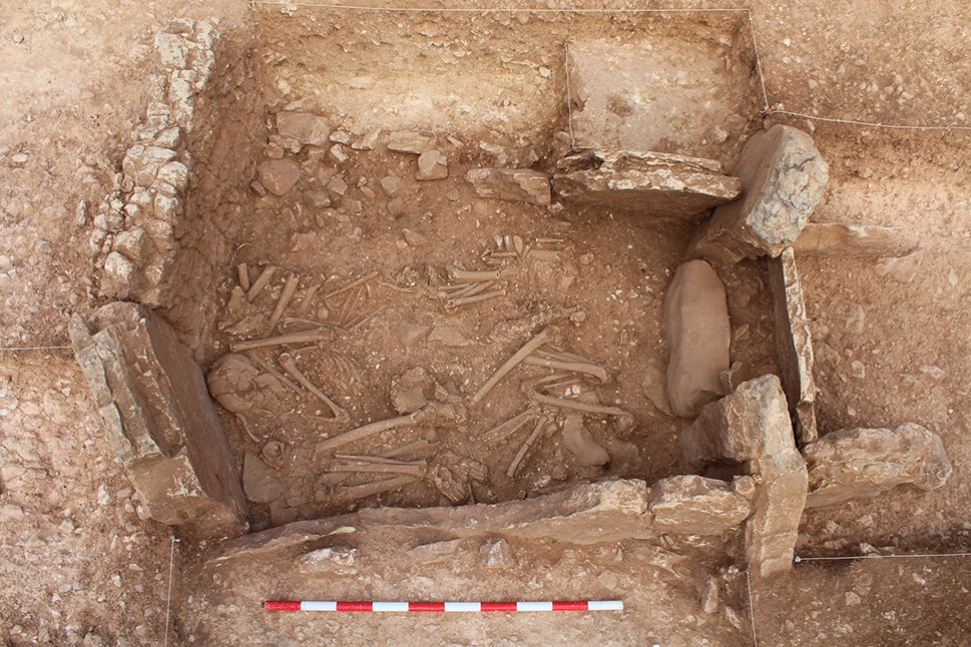
Human bone remains from Phase A from Tomb 11 with an articulated individual.

The megalithic cemetery of Panoría located in the south-east of the Iberian Peninsula belongs to a large European funerary tradition that in Spain began in the first decades of the IV millennium BC and, especially in the south-east, spanned until the end of the II millennium BC (Fig. 1). This site was discovered in 2012 and consists of at least 19 dolmens with polygonal or trapezoidal chambers and short corridors. In 2015 and 2019, nine tombs were excavated by the GEA research group from the University of Granada (www.webgea.es); four of them preserved largely undisturbed ritual deposits (Tombs 3, 10, 11 and 15). The tombs are aligned at regular intervals and most of them are orientated towards the sunrise at the equinox. Ritual and mortuary deposits consist of a complex multi-deposition of stratified and commingled human skeletal remains (Fig. 2). Although most of the bone remains appeared disarticulated, 22 individuals were found in anatomical position, ranging from complete bodies to specific anatomical parts. The latter were mainly upper or lower limbs resulting from previously buried individuals being moved during subsequent interments (Fig. 3). All these articulated skeletons were laid in a flexed position and, except in for three cases, bodies were placed on their left side. Twenty-one of the 22 individuals were oriented from west to east and aligned with the main axis of the funerary chambers^10,11, 29, 31–34^.

**Fig. 4:**
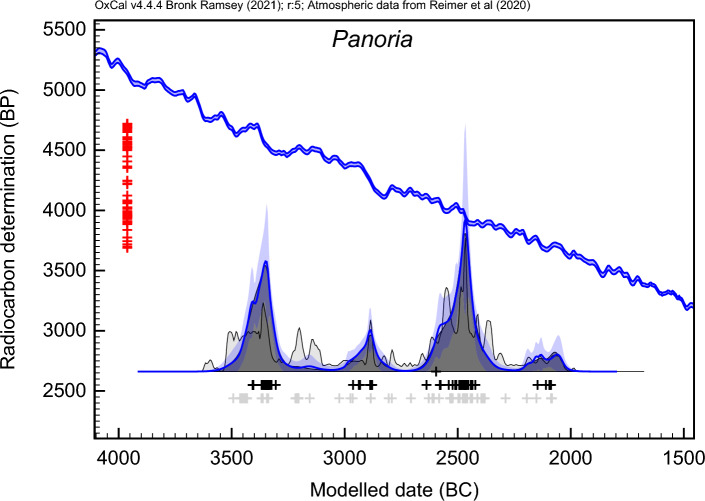
KDE-modelled distribution of all radiocarbon dates from Panoría cemetery (blue line). Radiocarbon measurements appear in red, the IntCal20 calibration curve in blue and the summed distribution in grey. Calibrated and modelled ages appear as grey and black crosses respectively.

In most of the Panoría tombs, the mortuary deposits appear to be separated into two distinct phases of ritual activity (Phases A and B). To better understand the timing of these multi-depositional burials, special attention was paid to their chronology, discussed in-depth elsewhere^10,32, 33^. For this purpose, a radiocarbon (^14^C) series of 73 dates belonging to human bones samples was produced. This chronological series, modelled in a Bayesian framework^35,36^, shows an earliest phase of funerary depositions (Phase B) dated to the second half of the 4th millennium cal BC (Fig. 4)^33^. Ritual activity began in most of the tombs around the 36th or 35th centuries cal BC. Nevertheless, mortuary activities reached their peak in the 34th century cal BC, with a brief resumption event in the twenty-ninth century cal BC. This primary ritual use spans three to six generations^33^. Around the twenty-ninth century cal BC, ritual practices ceased at most of the Panoría dolmens. After a hiatus of several centuries, megalithic tombs were reused, beginning a new period of funerary rituals in the first decades of the twenty-fifth century cal BC (Phase A). Funerary depositions were concentrated principally in the twenty-fifth and twenty-first centuries cal BC, with a few events spanning very few decades, approximately over one to four generations^33^. Funerary activity ended in the last century of the 3^rd^ millennium cal BC, coinciding chronologically with the development of the local Early Bronze Age known as Argaric Culture^37^.

Therefore, the Panoría cemetery presents a punctuated pattern of use with four peaks of ritual intensity dated in the thirty-fourth, twenty-ninth, twenty-fifth and twenty-first centuries and separated into two phases of mortuary practices (A and B)^33^. These short-lived events separated by periods of low or no funerary activity are highly consistent with the chronological models of a growing number of well-known European megalithic monuments, in which funerary activity was concentrated in brief periods spanning not more than several decades or even years^38–46^. The Panoría cemetery joins this European trend, producing a unique fine-grained chronology in Iberia that breaks down the traditional long chronological phases into shorter periods.

## Materials and methods

### Osteological analysis

The Panoría skeletal assemblage encompasses 57,872 bone fragments: 1200 teeth and 45 dental roots^11,30^. Detailed archaeological excavation methods (including systematic sieving) were applied, allowing us to confidently assert that the skeletal remains from each tomb were fully recovered^30^. However, the distribution of the bone assemblage by tomb shows an imbalance, with most of the skeletal remains concentrated in Tombs 3, 10, 11 and 15 (Table 1).

**Table 1:** Distribution of bone remains at the Panoría megalithic tombs. T: tomb; NISP: number of identified specimens. *Significant values are in bold.

Type of material	T3	T6	T7	T8	T10	T11	T15	T18	TOTAL
NISP	7940	29	1111	301	6981	16,180	10,372	809	57,872
Undetermined bone	3748	0	777	171	3793	2745	2780	135	
Tooth	281	5	23	13	352	329	188	9	1200
Root tooth	15	0	2	2	14	6	6	0	45
**TOTAL**	**11,984**	**34**	**1913**	**487**	**11,140**	**19,260**	**13,346**	**953**	**59,117**

Sex estimation of adults was mainly based on the dimorphic features of the pelvises and skulls. Due to the fragmentary state of the osteological remains, sex estimation relied on morphological traits of the skull, such as the nuchal crest, mastoid process, supraorbital margin, prominence of glabella, or mental eminence. The pelvic features of sexual dimorphism, such as the greater sciatic notch, acetabulum or preauricular sulcus, were only used in a few cases^5,47, 48^. In addition to osteological traits, the contextual archaeological information from each tomb was considered to ensure that the same individual was not identified twice^30^.

Age at death in adults was estimated using different methods: cranial suture closure^49^, morphological changes of the auricular surface^50^ and, despite the methodological limitations, dental attrition^51–53^. In non-adults, tooth development^54^ –crown formation, eruption and root completion– was prioritised over the development of long bone diaphysis and epiphyseal union^5,47, 55, 56^.

### Amelogenin peptide analysis

Sex estimation based on the sexually dimorphic amelogenin peptides in human tooth enamel is truly relevant when it comes to non-adult individuals whose morphology is not fully developed to show sexual dimorphism^16,17^. Moreover, dental enamel is resistant to diagenetic changes and can serve as an archive to decipher the chromosomal sex of individuals through the application of ultra-high performance chromatography tandem mass spectrometry (UHPLC-MS/MS).

Seven non-adult samples from the earliest ritual activity phase (Phase B) at the Panoría cemetery were analysed. Differences in tooth development in the case of Tomb 15 and the same tooth in the case of Tombs 3 and 11 were the criteria used to ensure that no individual was analysed twice. Sample preparation followed previous protocols^16,17^, extracting human enamel from each tooth crown by acid etching from a surface area of 2 × 2 mm.

Peptide standards, including Amelogenin Y1, Amelogenin Y2, Amelogenin Y3, Amelogenin X1 and Amelogenin X2, were injected into the LC–MS system at a concentration of 100 pg/µL to acquire specific retention times, peak shape abnormalities, exact m/z ratios and fragmentation patterns. The chromatography was optimised based on the elution times of these five peptides. Samples were reconstituted with 3 µL of 30% aqueous formic acid containing 10 fmol/µl peptide standards (Glu1-Fibrinopeptide B, M28, HK0, and HK1) and diluted with 17 µL LC–MS grade H_2_O.

Analysis was conducted using a 1290 Infinity II LC System (Agilent Technologies) coupled to an Orbitrap Exploris 480 mass spectrometer (Thermo Fisher Scientific) by an Optamax NG H-ESI (Thermo Fisher Scientific) as the ionisation source. 5 µl from each sample was injected into an XB-C18 (100 × 2.1 mm; 1.7 µm) column using H_2_O with 0.2% formic acid as solvent A and methanol with 0.2% formic acid as solvent B and a flowrate of 500 µl/min was applied. The gradient began at 20% B and increased to 40% B within 1.7 min. Peptides were analysed in + ESI mode with a resolution of 60,000 FWHM at m/z 200 in both MS1 and MS2, while the scan range in MS1 was set to 420–1400 m/z and MS2 was triggered with a data dependent top 2 approach.

Peak integration was performed using the Skyline software (Table 2). Peaks exceeding predefined cut-offs (Table 3) were integrated and recorded (Table 4). Samples that could not be determined using the automated Skyline pipeline were evaluated manually.

**Table 2:** Transition list of the selected gender specific peptides.

Molecule Name	Precursor m/z	Precursor Adduct	Precursor Charge	Retention time
AMELOGENIN-Y1	432.2257	[M + 2H]	2	1.59
AMELOGENIN-Y2	440.2233	[M + 2H]	2	1.21
AMELOGENIN-Y3	483.7393	[M + 2H]	2	1.19
AMELOGENIN-X1	540.2796	[M + 2H]	2	1.75
AMELOGENIN-X2	568.7903	[M + 2H]	2	1.64

**Table 3:** Characteristics of the respective peptide signals as well as the defined cut-off values during data evaluation.

Peptide gender	Peptide	Characteristics	Cut-off
Male	AMELOGENIN-Y1	Least sensitive and selective	1.5E5
Male	AMELOGENIN-Y2	Most sensitive and selective for male identification	1E6
Male	AMELOGENIN-Y3	Sensitive	1E5
Female	AMELOGENIN-X1	Sensitive and selective	1.5E5
Female	AMELOGENIN-X2	Most sensitive and selective for female identification	1E6

**Table 4:** Peak Areas.

AnChem ID	ID Peptide	1.59 AMELOGENIN-Y 1 (432.2257 m/z)	1.21 AMELOGENIN-Y 2 (440.2233 m/z)	1.19 AMELOGENIN-Y 3 (483.7393 m/z)	1.75 AMELOGENIN-X 1 (540.2796 m/z)	1.64 AMELOGENIN-X 2 (568.7903 m/z)	Ratio AMEL-X 1 / AMEL-Y 2	Peptide predicted sex
23-PEP-1628	Panoría 1	0	0	0	6.7E7	3.9E7	–	Female
23-PEP-1629	Panoría 2	0	0	0	4.0E7	2.3E7	–	Female
23-PEP-1630	Panoría 3	6.6E7	13.4E7	1.7E7	10.5E7	5.4E7	0.8	Male
23-PEP-1631	Panoría 4	0	4.1E6	0	20.1E7	9.5E7	48.6	Female
23-PEP-1632	Panoría 5	0	7.8E6	0	38.9E7	20.4E7	50.0	Female
23-PEP-1633	Panoría 6	0	0	0	4.5E7	2.3E7	–	Female
23-PEP-1634	Panoría 7	0	0	0	2.4E7	1.4E7	–	Female

The presence of Amelogenin Y and Amelogenin X indicated male samples, while the absence of Amelogenin Y and the presence of Amelogenin X indicated female samples. This ratio of Amelogenin X1/Amelogenin Y2 in male samples should be approximately 1/1. To validate an identified male sample, this ratio must not exceed 10/1, which may indicate a carryover effect causing an Amelogenin Y2 peak. The mass spectrometry proteomics data were deposited with the ProteomeXchange Consortium^57^ (http://proteomecentral.proteomexchange.org) via the PRIDE partner repository^58^ (Webpage: PXD052375, with the dataset identifier PXD052375 and *token: HeOAZXJXzilv).*

### Ancient DNA analyses

Molecular sex estimation was performed on 15 individuals from Tomb 10. To ensure that no individual was analysed twice, Tooth 45 was selected in all cases. Ancient DNA was obtained from dental roots at the Palaeogenomics Laboratory of La Laguna University (Canary Islands, Spain). All the processing was performed taking strict measures to avoid any contamination with modern DNA. The cementum from the root was pulverised using a bone mill to obtain a fine powder. Ancient DNA was extracted from the tooth powder using a silica-based protocol^59^ and then built into double-stranded libraries^60^. The ancient DNA libraries were sequenced on an Illumina NextSeq 500 platform at Sistemas Genómicos S.L. (Valencia, Spain), using a 2 × 75 bp paired-end protocol toobtain approximately 5 million reads per sample. The reads were trimmed and adapters removed using AdapterRemoval v2.1.7^61^ and then mapped to the human reference genome (hg19) using BWA v0.7.1^62^. Low quality (MAPQ < 25) and duplicate reads were removed using SAMtools v1.14^63^. Post-mortem damage was assessed in the ancient samples using MapDamage v2.2.1^64^ to identify DNA degradation patterns, including fragmentation and cytosine deamination at the molecule ends.

Molecular sex was determined using the RY estimate proposed by Skoglund et al.^19^ based on the ratio of sequences aligning to the X and Y chromosomes. Although the authors determined that molecular sex can be confidently identified from 100,000 genome-wide sequences, just ~ 10,000 sequences can provide a less confident but still accurate estimate in many cases^19^. Based on that and given the low conservation level of DNA at the Panoría site, we also considered the RY estimate for individuals down to 10,000 reads. To confirm our results, we performed sex estimation using the method proposed by Mittnick et al.^20^ on the same individuals. The RX method relies on comparing of the X chromosome coverage to that of the autosomes, in such a way that RX is expected to be ~ 0.5 for males and ~ 1.0 for females. The ancient DNA sequence data generated in this study are available through the European Nucleotide Archive (PRJEB75432).

### Sex ratio analysis

The sex ratio (SR) is the principal measure of sex composition. The SR formula is Pm/Pf × 100, where Pm is the number of males and Pf is the number of females^65^. The result, which can be formulated in hundreds or units, represents the number of males per female. In modern societies, SR ranges from 0.95:1 to 1.02:1 (M:F), with exceptions resulting from heavy warfare losses or immigration. Outside of the range 0.9:1 and 1.05:1 (M:F), the sex ratio is considered to be an extreme value^65^.

## Results

### Osteological sex identification

At Panoría cemetery, the minimum number of individuals amounts to 91, of which only 27 adult individuals were sexed (Table 5). This means that the 71% are estimated to be sexually “indeterminate”. The most accurate sex estimation that combines dimorphic features of the pelvis and skull could only be established in 11% of the individuals. Most of them were sexed based solely on dimorphic traits of the skulls (66.7%) followed by pelvic features (18.5%). Of the 27 individuals, 15 were females or probable females and 12 were males or probable males, which means a sex ratio of 0.8:1 (M:F). If probable males and females are removed, the sex ratio is 0.85:1 (M:F). If we consider that in human societies natural sex ratios range from 0.95:1 to 1.02:1 (M:F)^65^, the Panoría population shows an imbalance in favour of females.

**Table 5:** Biological sex estimation based on genetic, proteomic and osteological analyses at the Panoría cemetery. M: male; PM: probably male; F: female; PF: probably female.

Tomb	Phase	Method	Sex	Age at death	Radiocarbon age (BP)	Calibrate date (68% confidence) cal BC	Calibrate date (95% confidence) cal BC
3	Phase A	Osteology	M	Adult (26–40)	3985 ± 24	2565–2470 BC	2570–2465 BC
3	Phase A	Osteology	M	Adult (41–60)	3838 ± 24	2340–2210 BC	2450–2200 BC
3	Phase A	Osteology	F	Adult (26–40)	3969 ± 25	2560–2465 BC	2575–2350 BC
3	Phase B	Osteology	PF	Adult (26–40)	x	x	x
3	Phase B	Osteology	PF	Adult (41–60)	x	x	x
3	Phase B	Osteology	F	Adult (26–40)	x	x	x
3	Phase B	Osteology	PM	Adult	x	x	x
3	Phase B	Proteomics	F	Non-adult (7,5–8,5)	4705 ± 23	3520–3380 BC	3620–3375 BC
3	Phase B	Proteomics	F	Non-adult (16,5)	4719 ± 25	3605–3380 BC	3625–3380 BC
7	x	Osteology	PM	Adult	x	x	x
10	Phase A	Osteology	PM	Adult (41–60)	3899 ± 24	2465–2345 BC	2470–2300 BC
10	Phase A	Osteology	M	Adult (26–40)	x	x	x
10	Phase A	Osteology	F	Adult (18–25)	x	x	x
10	Phase A	Osteology	PF	Adult	x	x	x
10	Phase A	aDNA	PF	Adult	4019 ± 34	2570–2490 BC	2620–2470 BC
10	Phase A	aDNA	F	Adult	4026 ± 34	2580–2490 BC	2830–2470 BC
10	Phase A	aDNA	F	Adult	4025 ± 32	2580–2490 BC	2623–2470 BC
10	Phase A	aDNA	F	Adult	4013 ± 34	2570–2490 BC	2620–2470 BC
10	Phase B	aDNA	F	Adult	4072 ± 34	2830–2500 BC	2860–2490 BC
10	Phase A	aDNA	PM	Adult	3958 ± 34	2570–2410 BC	2570–2350 BC
10	Phase A	aDNA	PF	Adult	4059 ± 24	2625–2495 BC	2835–2490 BC
10	Phase B	aDNA	PM	Adult	4077 ± 24	2835–2570 BC	2850–2495 BC
10	Phase B	aDNA	M	Adult	4074 ± 21	2835–2570 BC	2840–2495 BC
10	Phase B	aDNA	M	Adult	4074 ± 24	2835–2570 BC	2850–2490 BC
11	Phase A	Osteology	F	Adult (41–60)	3689 ± 29	2140–2030 BC	2200–1970 BC
11	Phase A	Osteology	F	Adult (26–40)	3907 ± 29	2465–2350 BC	2470–2300 BC
11	Phase A	Osteology	M	Adult (26–40)	3775 ± 29	2280–2140 BC	2290–2050 BC
11	Phase A	Osteology	PF	Adult	3973 ± 29	2570–2465 BC	2580–2350 BC
11	Phase A	Osteology	PM	Adult	3951 ± 29	2560–2350 BC	2570–2340 BC
11	Phase A	Osteology	F	Adult (41–60)	3929 ± 29	2470–2350 BC	2560–2300 BC
11	Phase B	Osteology	PF	Adult (41–60)	x	X	x
11	Phase B	Osteology	PF	Adult	x	X	x
11	Phase B	Proteomics	M	Non-adult (7,5–8,5)	x	X	x
11	Phase B	Proteomics	F	Non-adult (9,5–11,5)	x	X	x
15	Phase B	Osteology	M	Adult (41–60)	4589 ± 28	3490–3350 BC	3500–3120 BC
15	Phase B	Osteology	PM	Adult (18–25)	4247 ± 28	2910–2880 BC	2910–2710 BC
15	Phase B	Osteology	PF	Adult (41–60)	4548 ± 28	3370–3120 BC	3370–3100 BC
15	Phase B	Osteology	M	Adult (41–60)	4570 ± 28	3370–3140 BC	3490–3110 BC
15	Phase B	Osteology	PF	Adult (18–25)	4567 ± 28	3370–3140 BC	3490–3110 BC
15	Phase B	Osteology	PM	Adult (18–25)	4651 ± 29	3500–3370 BC	3520–3370 BC
15	Phase B	Osteology	F	Adult (18–25)	x	x	x
15	Phase B	Proteomics	F	Non-adult (12,5–13,5)	4519 ± 28	3350–3110 BC	3360–3100 BC
15	Phase B	Proteomics	F	Non-adult (11,5–12,5)	4554 ± 28	3370–3130 BC	3480–3100 BC
15	Phase B	Proteomics	F	Non-adult (16,5)	4407 ± 29	3090–2930 BC	3310–2920 BC

### Peptide-based sexing

Peptide-based sexing provided reliable results for all analysed non-adults. The results showed that six out of seven individuals were compatible with the female sex and only one (Tomb 11) showed traces of Amelogenin Y, which was indicative of a male individual. According to these results, the non-adult population of Panoría shows an extreme imbalance in female representation, as the sex ratio is 0.16:1 (M:F). Furthermore, this sex profile is even more significant because male children tend to be more susceptible to disease than females^66–69^. In the absence of any kind of cultural selection, childhood mortality is expected to be higher in males than in females.

### Genetic-based sexing

DNA preservation for the Panoría cemetery is poor, with an average value of 0.801% endogenous DNA and a median value of 0.364%. The best-preserved samples are LLD-0069, LLD-0067 and LLD-0074 with endogenous DNA contents of 3.91%, 2.26% and 2.18%, respectively (Table S1). The remaining individuals show endogenous DNA percentages below 1%. Duplicates proportion has a mean value of 1.95% and a median of 1.18%, although some samples reach values of 7.14% (LLD-0063) and 6.50% (LLD-0075), reflecting again the poor DNA preservation.

All samples have evidence of post-mortem damage as expected from ancient DNA (Table S1). The endogenous DNA shows short average insert sizes due to fragmentation, with values between 31.1 and 37.9 pair bases (Fig. S1). Damage at the end of the molecules due to deamination ranges from 27 to 49% (Fig. S1). Due to low coverage, contamination estimation from either the mitochondrial or nuclear DNA was not possible.

**Fig. 5:**
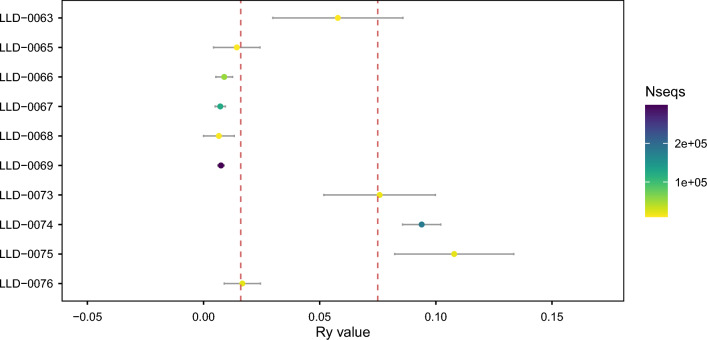
Molecular sex determination based on RY estimates for the Panoría individuals. RY estimate values are shown for each sample, including the 95% confidence intervals. Red lines indicate the CI upper bound for female assignation (0.016) and the CI lower bound for male assignation (0.077). Dot colours correspond to the amount of total endogenous reads used for RY estimation.

**Fig. 6:**
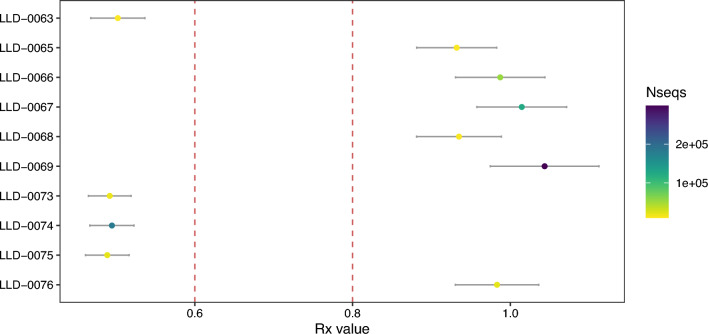
Molecular sex determination based on RX estimates for the Panoría individuals. RX estimate values are shown for each sample, including the 95% confidence intervals. Red lines indicate the CI upper bound for male assignation (0.6) and the CI lower bound for female assignation (0.8). Dot colours correspond to the amount of total endogenous reads used for RX estimation.

After mapping, only 3 out of 15 individuals reached the minimum of 100,000 reads for performing a confident molecular sex determination using RY (Table S1), accounting for a success rate of just 20%. However, when we considered a threshold of 10,000 reads, it was possible to sex a total of 10 individuals (66.7%) (Fig. 5). It is worth mentioning that all classifications were confirmed using the RX method (Table S1; Fig. 6). RY estimation results indicated that four individuals were female and two more were consistent with being female rather than male. Two individuals were classified as male and two were consistent with being male. Overall, the DNA analysis based on RY indicated that six individuals were female and four were male (Table S1). Uncertainty in the molecular sex determination is due in all cases to poor conservation and low endogenous DNA content (Fig. 5). For the two individuals with RY estimates consistent with being XX but not XY, LLD-0065 and LLD-0076, the number of total reads after filtering mapping to the human genome were around 11,800 and 21,900, respectively. For the individuals consistent with being males but not females, LLD-0071 and LLD-0073, these values were around 9700 and 18,000, respectively (Table S1; Fig. 5). All the individuals that were consistent with being male and female for RY were confirmed to belong to that sex using RX (Fig. 6). The sex ratio 0.66:1 (M:F) again emphasised an imbalance in favour of females. If individuals identified as consistent with male or female are removed, the sex ratio is even more extreme, 0.5:1 (M:F).

## Discussion

The osteological, proteomic and genomic sex estimation methods present different challenges and limitations. A recent comparison between these methods using a sample of 55 individuals from two ancestral Ohlone sites in Central California correctly identified the sex in 100% of the individuals using proteomics, in 91% using genomics (although with different degrees of reliability), and in 51% using osteological methods (24% only as possible sex-determination)^70^. Osteological examination appears to be the most limited method as appropriate morphological criteria are not always sufficiently preserved to estimate sex. Skeletal morphology may also include different intra and interobserver errors, as sexual dimorphism could be the result of biological, social and environmental interactions^71–74^.

Another important bias is the overestimation of males in bone collections that are characterised by poor preservation of the pelvis in comparison to the skull. Skeletal traits of the skull, such as the supraorbital ridges, mastoid processes and temporal and superior nuchal lines, tend to be classified as male, even in cases where they are moderately marked^75,76^. This frequently appears to be the case in skeletons from the Iberian Peninsula. According to a recent study based on a large database of 2,410 individuals from 62 prehistoric sites spanning approximately the 7th to the 3rd millennium, a strong male bias appears as a key aspect in most of the different cultural periods and analysed archaeological sites^77^. The first genetic and proteomic sex-based estimations conducted in Iberia clearly support this overestimation. Not only does the osteological male bias disappear in these recent studies^26^, but even the opposite pattern emerges as a sex ratio imbalance in favour of females^27,28,78^.

This is the case of the 44 different individuals sexed at the Panoría cemetery. Table 5 shows results from the three methods that provide sex estimations. Proteomic analysis of amelogenin provided sex estimates for the non-adult population (16%) and osteology a molecular sex for adults (84%). The morphology-based estimate shows a sex ratio of 0.8:1 (M:F) in female representation, which falls to 0.6:1 (M:F) when proteomic and genomic sex determination is considered. If probable males and females are not taken into account, the overall sex ratio drops to 0.5:1 (M:F), i.e. twice as many females as males. This is even more extreme in the non-adult population, where the sex ratio is 0.16 (M:F).

This sex imbalance can also be explored in relation to the two main periods of use identified at the Panoría cemetery. Of the 44 sexed individuals, 43 can be associated with one of the two phases: 24 with the earliest (Phase B) and 19 with the most recent (Phase A) (Table 5). In both cases, the imbalance in favour of females stands out with sex ratios of 0.6:1 (M:F) and 0.58:1 (M:F), respectively. If the possible males and females are removed, the sex ratios are 0.55:1 (M:F) and 0.50:1 (M:F), a very similar sex imbalance. The main difference between periods appears among the non-adults, with the extreme imbalance concentrated in the earliest phase of mortuary activity. The imbalance in female representation is also a key feature of the four tombs with well-preserved ritual deposits (Tombs 3, 10, 11 and 15). Sex ratios range from 0.75:1(M:F) in Tomb 10 to 0.42:1(M:F) in Tomb 11, in all cases below the threshold considered to be the natural sex ratio of 0.95:1 to 1.02:1 (M:F)^65^.

The male/female ratio at Panoría shows a strong bias in favour of females, regardless of the criterion considered: sex ratio by tomb, chronological period, method of sex estimation or age group.On the Iberian Peninsula, there are only a few cases of megalithic populations that support a clear female sex bias. Montelirio (south-western Iberia), a *tholos*-type tomb, contained the remains of 20 adult individuals in the main funerary chamber, 15 of them female or probably female and the remaining five classified as “indeterminate”^79^. In the La Rioja region (northern Iberian Peninsula), the comparison between megalithic tombs and burial caves shows a distinctive pattern: a male sex bias in megalithic monuments against a female bias in caves, with sex ratios of 1.33:1 and 0.45:1 (M:F), respectively^80^. The Late Neolithic necropolis of Escoural cave in southern Portugal also represents a good reference for comparison. The proteomic analysis of a population of 36 individuals shows a sex ratio of 0.5:1 (M:F) favouring of females^27^, similar to the female-related bias found at Panoría.

## Conclusions

In the context of megalithic societies, Panoría stands out as the necropolis with the largest number of sex estimations in Iberia, including non-adults for the first time. The Panoría population shows an extremely unbalanced demographic pattern favouring females that cannot be explained by natural dynamics or extraordinary and unpredictable events. Different aspects support this statement. Regardless of their chronology, the unbalanced sex ratio is a key feature of the different phases of funerary activity that are concentrated in four peaks of ritual intensity dated in the thirty-fourth, twenty-ninth, twenty-fifth and twenty-first centuries. Furthermore, sex bias can be found in all tombs and different age categories. The overrepresentation of females can thus be considered a temporally persistent social pattern characteristic of the social groups that buried their dead in the different Panoría dolmens.

If sex bias is a social pattern, what are the social reasons for this over-representation of females in funerary rituals? Why is the sex bias so persistent in the megalithic societies at Panoría? The sex bias found at Panoría would indicate an emphasis on selecting and depositing females. This suggests that the mortuary practices of the Panoría necropolis could have been mainly based on matrilineal descent. It seems that funerary depositions were selective, prioritising females to be buried in the megalithic tombs. It is possible that lineage daughters stayed with the kin group while sons left to join other kin groups (male exogamy). The extreme sex bias found in non-adults at Panoría with a sex ratio of 0.16:1 (M:F) would support this possibility. However, the hypothesis of matrilineal descendent, where females are prioritised, needs further corroboration. Establishing the genetic relationships between individuals is paramount for testing this interpretive proposal as it has been proved at major megalithic sites in Ireland and Britain^81-83^. The overrepresentation of females among the Panoría population is probably indicatesa female-centred social structure, in which sex and/or gender would have influenced funerary rites and cultural traditions.

## Supplementary Information

Supplementary Information 1.

Supplementary Information 2.

Supplementary Information 3.

Supplementary Information 4.

## Data Availability

The mass spectrometry proteomics data have been deposited to the ProteomeXchange Consortium^53^ (http://proteomecentral.proteomexchange.org) via the PRIDE partner repository^49^. Project accession: PXD052375. Project Not applicable. Reviewer access details: Unique link: https://www.ebi.ac.uk/pride/archive. Project accession: PXD052375. Token: HeOAZXJXzilv. Alternatively, reviewer can access the dataset by logging into the PRIDE website using the following account details: Username: reviewer_pxd052375@ebi.ac.uk. Password: BzMUcO1EKQ2k. Ancient DNA sequence data generated in this study are available through the European Nucleotide Archive (PRJEB75432) in the intext of the manuscript. Contact person: rfregel@ull.edu.es. The orthophotography was created by G.J.A. using a drone equipment (DJI Inspire-1) that incorporated one FC350 camera with a resolution of 4000 × 3000 pixels and a field of view (FOV) of 94° for a 3.61 mm lens. The camera included a 1/2.3 inch Sony Exmor CMOS sensor. Dron images were processed with a SfM-based technology Agisoft-photoscan pro® 2.0.
