# Author Correction: Female sex bias in Iberian megalithic societies through bioarchaeology, aDNA and proteomics

**DOI:** 10.1038/s41598-024-78427-x

**Published:** 2024-11-14

**Authors:** Marta Díaz-Zorita Bonilla, Gonzalo Aranda Jiménez, Margarita Sánchez Romero, Rosa Fregel, Katharina Rebay-Salisbury, Fabian Kanz, Miriam Vílchez Suárez, Sonia Robles Carrasco, Paula Becerra Fuello, Alejandra C. Ordóñez, Michael Wolf, Javier González Serrano, Lara Milesi García

**Affiliations:** 1 Institute for Pre- and Protohistory and Medieval Archaeology, University of Tübingen, Tübingen, Germany; 2 Department of Prehistory and Archaeology, University of Granada, Granada, Spain; 3 Department of Biochemistry, Microbiology, Cell Biology and Genetics, Universidad de La Laguna, San Cristóbal de La Laguna, Spain; 4 Department of Prehistoric and Historical Archaeology, University of Vienna, Vienna, Austria; 5 Austrian Academy of Sciences, Vienna, Austria; 6 Center for Forensic Medicine, Medical University of Vienna, Vienna, Austria; 7 Department of Historical Sciences, University of Las Palmas de Gran Canaria, Las Palmas de Gran Canaria, Spain; 8 Department of Analytical Chemistry, University of Vienna, Vienna, Austria; 9 Department of Historical Sciences, University of Malaga, Malaga, Spain

Correction to: *Scientific Reports *10.1038/s41598-024-72148-x, published online 23 September 2024

The original version of this Article contained errors in the name of authors Marta Díaz-Zorita Bonilla, Gonzalo Aranda Jiménez, Margarita Sánchez Romero, Rosa Fregel, Katharina Rebay-Salisbury, Fabian Kanz, Miriam Vílchez Suárez, Sonia Robles Carrasco, Paula Becerra Fuello, Alejandra C. Ordóñez, Michael Wolf, Javier González Serrano & Lara Milesi García.

The original Article has been corrected.

