# Author Correction: Female sex bias in Iberian megalithic societies through bioarchaeology, aDNA and proteomics

**DOI:** 10.1038/s41598-025-98345-w

**Published:** 2025-04-28

**Authors:** Marta Díaz-Zorita Bonilla, Gonzalo Aranda Jiménez, Margarita Sánchez Romero, Rosa Fregel, Katharina Rebay-Salisbury, Fabian Kanz, Miriam Vílchez Suárez, Sonia Robles Carrasco, Paula Becerra Fuello, Alejandra C. Ordónez, Michael Wolf, Javier González Serrano, Lara Milesi García

**Affiliations:** 1 Institute for Pre- and Protohistory and Medieval Archaeology, University of Tübingen, Tübingen, Germany; 2 Department of Prehistory and Archaeology, University of Granada, Granada, Spain; 3 Department of Biochemistry, Microbiology, Cell Biology and Genetics, Universidad de La Laguna, San Cristóbal de La Laguna, Spain; 4 Department of Prehistoric and Historical Archaeology, University of Vienna, Vienna, Austria; 5 Austrian Academy of Sciences, Vienna, Austria; 6 Center for Forensic Medicine, Medical University of Vienna, Vienna, Austria; 7 Department of Historical Sciences, University of Las Palmas de Gran Canaria, Las Palmas de Gran Canaria, Spain; 8 Department of Analytical Chemistry, University of Vienna, Vienna, Austria; 9 Department of Historical Sciences, University of Malaga, Malaga, Spain

Correction to: *Scientific Reports* 10.1038/s41598-024-72148-x, published online 23 September 2024

The original version of this Article contained an error in the spelling of the author Gonzalo Aranda Jiménez which was incorrectly given as Gonzalo Jiménez Aranda.

The original Article has been corrected.

