# Supplementary figures and images for: Female sex bias in Iberian megalithic societies through bioarchaeology, aDNA and proteomics
Source: Sci Rep. 2024 Sep 23;14:21818. doi: 10.1038/s41598-024-72148-x (PMC11420231; doi:10.1038/s41598-024-72148-x)

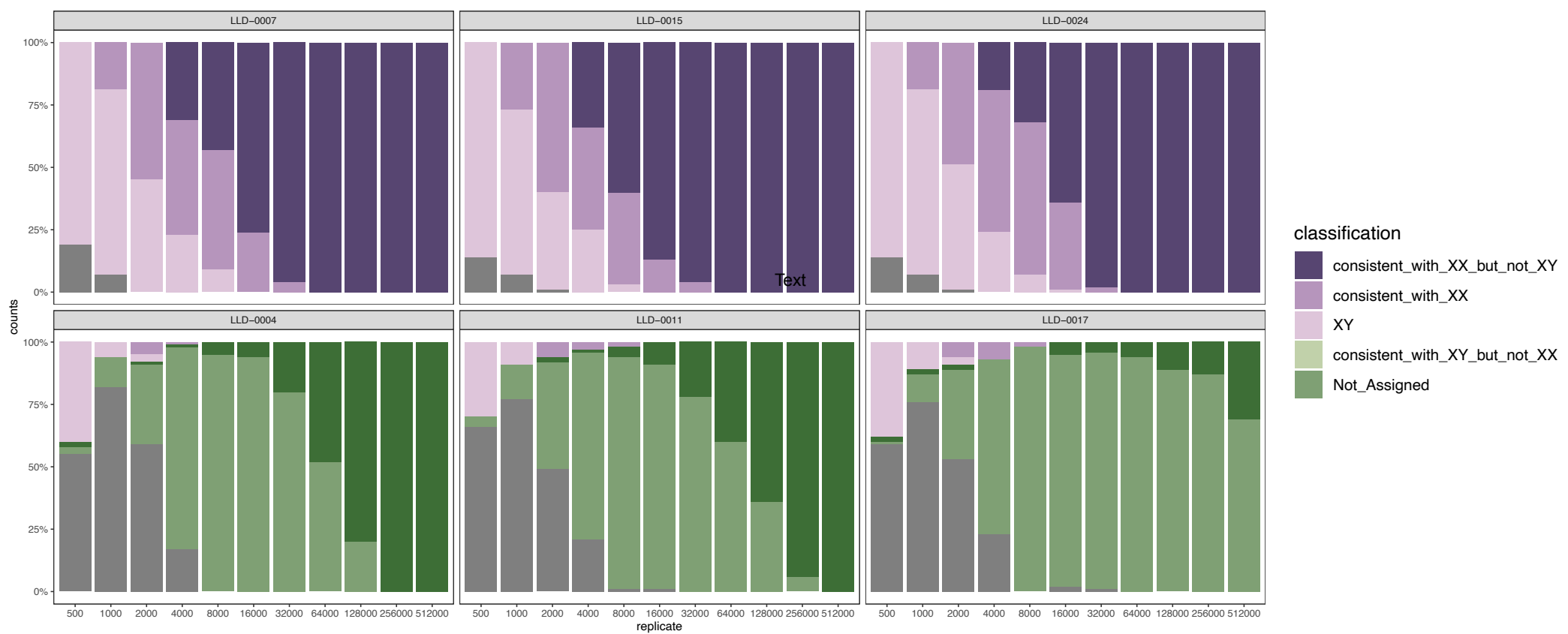

Supplement: Supplementary file 1 — Supplementary Information 1. [file 41598_2024_72148_MOESM1_ESM.pdf]
