# Supplementary material for: Female sex bias in Iberian megalithic societies through bioarchaeology, aDNA and proteomics
Source: Sci Rep. 2024 Sep 23;14:21818. doi: 10.1038/s41598-024-72148-x (PMC11420231; doi:10.1038/s41598-024-72148-x)
Supplement: Supplementary file 2 — Supplementary Information 2. [file 41598_2024_72148_MOESM2_ESM.docx]

**Supplementary Legends**

Table S1 - Summary of ancient DNA results for the Panoría individuals.

Individuals excluded from the study due to low endogenous content are shown in grey.

Figure S1 – DNA damage pattern (A) and insert length distribution (B) plots for the Panoría individuals
