# Supplementary material for: Female sex bias in Iberian megalithic societies through bioarchaeology, aDNA and proteomics
Source: Sci Rep. 2024 Sep 23;14:21818. doi: 10.1038/s41598-024-72148-x (PMC11420231; doi:10.1038/s41598-024-72148-x)
Supplement: Supplementary file 3 — Supplementary Information 3. [file 41598_2024_72148_MOESM3_ESM.pdf]

| Library ID | Laboratory ID | Sample ID | Sequenced reads | Mapped reads | Q25 reads | Unique reads |
|---|---|---|---|---|---|---|
| LLD-0063 | PGULL-1257 | PAN20 | 3.826.788 | 18.188 | 12.227 | 11.294 |
| LLD-0064 | PGULL-1258 | PAN21 | 3.841.382 | 12.445 | 8.396 | 8.286 |
| LLD-0065 | PGULL-1259 | PAN22 | 2.864.942 | 18.372 | 12.683 | 12.523 |
| LLD-0066 | PGULL-1260 | PAN23 | 5.660.104 | 84.268 | 58.468 | 57.835 |
| **LLD-0067** | **PGULL-1261** | **PAN24** | **5.544.462** | **184.656** | **131.037** | **129.759** |
| LLD-0068 | PGULL-1262 | PAN25 | 5.509.811 | 20.009 | 14.250 | 14.101 |
| **LLD-0069** | **PGULL-1263** | **PAN26** | **7.733.050** | **441.622** | **314.553** | **311.045** |
| LLD-0070 | PGULL-1264 | PAN27 | 3.030.157 | 1.699 | 1.237 | 1.216 |
| LLD-0071 | PGULL-1265 | PAN28 | 4.184.322 | 15.251 | 10.531 | 10.346 |
| LLD-0072 | PGULL-1266 | PAN29 | 2.803.185 | 1.078 | 829 | 818 |
| LLD-0073 | PGULL-1267 | PAN362274 | 4.655.660 | 27.967 | 19.128 | 18.943 |
| **LLD-0074** | **PGULL-1268** | **PAN362297** | **8.385.104** | **280.741** | **190.630** | **188.097** |
| LLD-0075 | PGULL-1269 | PAN362275 | 5.625.589 | 35.542 | 23.954 | 22.329 |
| LLD-0076 | PGULL-1270 | PAN362291 | 9.019.027 | 33.975 | 23.673 | 23.317 |
| LLD-0077 | PGULL-1271 | PAN3622105 | 458.588 | 3.543 | 2.319 | 2.277 |

| Endogenous DNA | Duplicates | Damage | Average insert size (bp) | Total human reads for RY and RX estimates (Q30) | Cromosome X reads |
|---|---|---|---|---|---|
| 0,295 | 7,63% | 27,0% | 35,78 | 10.479 | 261 |
| 0,216 | 1,31% | 31,0% | 35,37 | 7.608 | NA |
| 0,437 | 1,26% | 35,5% | 35,92 | 11.772 | 550 |
| 1,022 | 1,08% | 40,9% | 36,71 | 54.825 | 2.670 |
| **2,340** | **0,98%** | **37,0%** | **37,75** | **124.702** | **6.219** |
| 0,256 | 1,05% | 37,1% | 36,44 | 13.034 | 603 |
| **4,022** | **1,12%** | **36,9%** | **37,91** | **298.906** | **15.140** |
| 0,040 | 1,70% | NA | 32,90 | 922 | NA |
| 0,247 | 1,76% | 29,5% | 36,81 | 9.673 | NA |
| 0,029 | 1,33% | NA | 31,16 | 567 | NA |
| 0,407 | 0,97% | 31,5% | 36,39 | 17.992 | 451 |
| **2,243** | **1,33%** | **34,7%** | **37,50** | **181.361** | **4.516** |
| 0,397 | 6,78% | 37,3% | 37,54 | 20.996 | 521 |
| 0,259 | 1,50% | 32,3% | 36,20 | 21.858 | 1.062 |
| 0,497 | 1,81% | 49,0% | 33,40 | 2.152 | NA |

| Chromosome Y reads | Ry estimate | Ry SE | Ry 95% confidence inverval | Ry assignment |
|---|---|---|---|---|
| 16 | 0,0578 | 0,0140 | 0.0303-0.0852 | consistent with XY but not XX |
| NA | NA | NA | NA | NA |
| 8 | 0,0143 | 0,0050 | 0.0045-0.0242 | consistent with XX but not XY |
| 24 | 0,0089 | 0,0018 | 0.0054-0.0125 | XX |
| **45** | **0,0072** | **0,0011** | **0.0051-0.0093** | **XX** |
| 4 | 0,0066 | 0,0033 | 0.0002-0.013 | XX |
| **115** | **0,0075** | **0,0007** | **0.0062-0.0089** | **XX** |
| NA | NA | NA | NA | NA |
| NA | NA | NA | NA | NA |
| NA | NA | NA | NA | NA |
| 37 | 0,0758 | 0,0120 | 0.0523-0.0993 | consistent with XY but not XX |
| **468** | **0,0939** | **0,0041** | **0.0858-0.102** | **XY** |
| 63 | 0,1079 | 0,0128 | 0.0827-0.133 | XY |
| 18 | 0,0167 | 0,0039 | 0.009-0.0243 | consistent with XX but not XY |
| NA | NA | NA | NA | NA |

| Rx estimate | Rx SE | Rx 95% confidence inverval | p-value | Rx assignment |
|---|---|---|---|---|
| 0,5023 | 0,0175 | 0.4680-0.5367 | 3,06E-12 | XY |
| NA | NA | NA | NA | NA |
| 0,9320 | 0,0259 | 0.8812-0.9827 | 4,01E-14 | XX |
| 0,9872 | 0,0289 | 0.9305-1.0439 | 3,42E-16 | XX |
| **1,0145** | **0,0290** | **0.9576-1.0713** | **< 2.2e-16** | **XX** |
| 0,9349 | 0,0275 | 0.8811-0.9887 | 4,97E-16 | XX |
| **1,0435** | **0,0352** | **0.9745-1.1124** | **2,47E-16** | **XX** |
| NA | NA | NA | NA | NA |
| NA | NA | NA | NA | NA |
| NA | NA | NA | NA | NA |
| 0,4921 | 0,0138 | 0.4651-0.5192 | 7,35E-12 | XY |
| **0,4949** | **0,0143** | **0.4669-0.5228** | **1,79E-12** | **XY** |
| 0,4890 | 0,0141 | 0.4613-0.5166 | 6,78E-12 | XY |
| 0,9831 | 0,0270 | 0.9302-1.0360 | 2,68E-16 | XX |
| NA | NA | NA | NA | NA |
